# Enhanced Cocaine-Associated Contextual Learning in Female H/Rouen Mice Selectively Bred for Depressive-Like Behaviors: Molecular and Neuronal Correlates

**DOI:** 10.1093/ijnp/pyv022

**Published:** 2015-04-30

**Authors:** Virginie Rappeneau, Anne-Laure Morel, Malika El Yacoubi, Jean-Marie Vaugeois, Luc Denoroy, Anne Bérod

**Affiliations:** INSERM U1028/CNRS UMR5292, Lyon Neuroscience Research Center, Team Physiopathology of the neuronal network responsible for the sleep-wake cycle, Lyon, France (Ms Morel, Drs Rappeneau, El Yacoubi, and Bérod); University Claude Bernard Lyon 1, Lyon, France (Ms Morel, Drs Rappeneau, El Yacoubi, Denoroy, and Bérod); 2EA 4651-ABTE /ToxEMAC Rouen, France (Dr Vaugeois); University of Rouen, Rouen, France (Dr Vaugeois); INSERM U1028/CNRS UMR5292, Lyon Neuroscience Research Center, Team BioRaN, Lyon, France (Dr Denoroy).

**Keywords:** BDNF, cocaine, depression, H/Rouen mice, sex differences

## Abstract

**Background:**

Major depression has multiple comorbidities, in particular drug use disorders, which often lead to more severe and difficult-to-treat illnesses. However, the mechanisms linking these comorbidities remain largely unknown.

**Methods:**

We investigated how a depressive-like phenotype modulates cocaine-related behaviors using a genetic model of depression: the Helpless H/Rouen (H) mouse. We selected the H mouse line for its long immobility duration in the tail suspension test when compared to non-helpless (NH) and intermediate (I) mice. Since numerous studies revealed important sex differences in drug addiction and depression, we conducted behavioral experiments in both sexes.

**Results:**

All mice, regardless of phenotype or sex, developed a similar behavioral sensitization after 5 daily cocaine injections (10 mg/kg). Male H and NH mice exhibited similar cocaine-induced conditioned place preference scores that were only slightly higher than in I mice, whereas female H mice strikingly accrued much higher preferences for the cocaine-associated context than those of I and NH mice. Moreover, female H mice acquired cocaine-associated context learning much faster than I and NH mice, a facilitating effect that was associated to a rapid increase in striatal and accumbal brain-derived neurotrophic factor levels (BDNF; up to 35% 24 h after cocaine conditioning). Finally, when re-exposed to the previously cocaine-associated context, female H mice displayed greater Fos activation in the cingulate cortex, nucleus accumbens, and basolateral amygdala.

**Conclusions:**

Our data indicate that neurobiological mechanisms such as alterations in associative learning, striato-accumbal BDNF expression, and limbic-cortico-striatal circuit reactivity could mediate enhanced cocaine vulnerability in female depressive-like mice.

## Introduction

Major depressive disorder (hereafter referred to as depression) has multiple comorbidities, in particular substance use disorders (SUD), which often lead to more severe and difficult-to-treat illnesses (Davis et al., 2008; Pettinati et al., 2013). This is well demonstrated for psychostimulant drugs, such as cocaine. Compared to the general population, depressed patients display substantially higher rates of lifetime cocaine use. Additionally, cocaine abusers show a greater lifetime prevalence of depression (Conway et al., 2006). Much debate surrounds the question of whether overlapping neurobiological vulnerability factors contribute to the close intertwining of depression and SUD.

Increasing evidence in humans indicates that depressive states are likely determinants of cocaine use and abuse vulnerability (for review see Cheetham et al., 2010), a notion that is supported by preclinical studies. Rodent models of depression that have attempted to reproduce core components of this disorder through exposure of animals to early-life experiences or repeated stress in adulthood have generally established that depressive-like states increase cocaine rewarding effects and relapse in regard to self-administration and conditioned place preference (CPP) paradigms (Covington and Miczek, 2001, 2005; Nader et al., 2012; Bardo et al., 2013). However, depression is a complex and heterogeneous disorder in terms of its etiology and biology, in which both genetic and environmental factors play a fundamental role. In this regard, recent studies in rodents suggest that mechanisms that promote depressive symptoms in response to environmental stressors (“reactive” depression) may be distinct from those stemming from genetic predisposition (“endogenous” depression; Malki et al., 2014; see Krishnan and Nestler, 2008, for review). These findings emphasize the need for developing and comparing models of depression built on different strategies. Surprisingly, genetic rodent models of depression modeling stable core behavioral features have provided few insights into the underlying neurobiological correlates and their interactions with cocaine vulnerability.

Based on the aforementioned evidence, the current study sought to further investigate how a depressive-like phenotype modulates cocaine-related behaviors using a genetic mouse model of depression, the Helpless H/Rouen (H) mouse (El Yacoubi et al., 2003). H mice were selected for their long immobility duration in the Tail Suspension Test (TST), a behavioral expression of helplessness widely used for screening antidepressant drugs and modeling individual differences in the stress response (Steru et al., 1985). Not only does this mouse line markedly differ from non-helpless (NH) and intermediate (I) mice derived from the same colony in terms of immobility duration in the TST (short and intermediate duration for NH and I mice, respectively), it is also the only one of the three that displays several behavioral and neurochemical impairments that mimic those found in depression. Notably, H mice display anhedonia, exacerbated depressive- and anxiety-like behaviors, sleep disturbances, and positive responses to antidepressants (El Yacoubi et al., 2003, 2013; Popa et al., 2006).

Thus, we compared the depressive-like H mice to I mice, the control mouse line, and to NH mice, which can be seen as extreme with their non-helpless phenotype in terms of sensitivity to cocaine psychomotor effects and ability to associate a specific environmental context with cocaine rewarding effects in the CPP paradigm, a learned association that plays a crucial role in addiction. Previous animal and human studies revealed important sex differences in drug addiction (see Bobzean et al., 2014, for review) and depression (see Parker and Brotchie, 2010, for review). Therefore, we conducted the behavioral experiments in both sexes. We also explored the molecular mechanisms and anatomical pathways behind the enhanced cocaine-induced CPP observed in female H mice using brain-derived neurotrophic factor (BDNF) expression and Fos imaging. We examined the expression of BDNF that is an attractive candidate protein for its potential involvement in the co-occurrence of SUD in depression (Russo and Nestler, 2013). We used Fos-imaging to compare brain reactivity patterns in the three mouse lines during retrieval of the cocaine-associated contextual memory during the CPP test.

## Materials and Methods

### Animals

Mice selectively bred for high (H), intermediate (I), and low (NH) spontaneous helplessness in the Tail Suspension Test were derived from an original stock of albino CD1 mice (El Yacoubi et al., 2003, 2013). They were housed with same-sex littermates in groups of three to six under controlled conditions (21±1°C, 12h light cycle) and provided with water and a standard rodent diet (A04, Scientific Animal Food Engineering).

All experiments were performed with mice aged from 9 to 18 weeks and from generations S36-S43 for the extreme (NH, H) phenotypes and S18-S25 for the intermediate (I) phenotype. All animal procedures were conducted in strict accordance with ethical principles and guidelines on the care and use of laboratory animals adopted by the European Community (law 86/609/CEE). Authorization to conduct experiments on living animals was obtained from the Direction Départementale des Services Vétérinaires du Rhône (authorization number 69266242).

### Drugs

Cocaine hydrochloride (COOPER, Cooperation Pharma ceutique Française) dissolved in sterile saline solution (0.9% NaCl) was injected i.p. into mice at different concentrations in a volume of 10 ml/kg.

### Behavioral Assessments in Mice

#### Tail Suspension Test

As part of the breeding process, 6-week-old NH, I, and H mice were submitted to the TST as previously described (El Yacoubi et al., 2003, 2013). The chosen selection criteria, which were the same at each generation, were a high immobility score (>115 s) for H, a low immobility score (<35 s) for NH, and intermediate scores (between 35 and 115 s) for I mice. In typical experiments in female animals, immobility scores were 278 s ± 11.95 (ranging from 213 to 331 s) for H mice, 97 s ± 8.92 (ranging from 38 to 113 s) for I mice, and 3 s ± 0.33 (ranging from 1 to 5 s) for NH mice. The immobility scores in I mice correspond to those of 70% of the original stock of CD1 mice (Vaugeois et al., 1997).

#### General Procedure for Behavioral Experiments

Prior to any behavioral experiments, experimentally naive mice were individually housed for 4 days and mice remained single-housed across the behavioral experiments. On the day of the experiment, animals were brought to the test room 30 min before the start of the experiment for habituation and to reduce stress. Independent groups of mice were used for each behavioral test and all experiments were performed between 10:00 and 18:00 hours. Male and female mice were tested separately in the same behavioral apparatus.

#### Cocaine-Induced Locomotor Activity

Locomotor activity was monitored in a four-chamber actimeter (50 x 50 x 45 cm) with a video-tracking device (Viewpoint) detecting the location of the animal and monitoring ambulatory activity. The actimeter was located inside an isolated dark room. Mice were first habituated to the locomotor chamber for 120 min. Then, they received an intraperitoneal injection of cocaine (1, 3, 10, 20, or 30 mg/kg) or saline and were immediately placed back in the chamber for 45 min. Since a single exposure to cocaine can induce a persistent locomotor sensitization to this drug (Valjent et al., 2010), each mouse received only one dose of cocaine. The median effective doses (ED_50_) values were calculated using nonlinear regression assuming sigmoidal dose-responses with variable slopes (OriginLab 7.5).

#### Cocaine-Induced Behavioral Sensitization

For induction of behavioral sensitization, mice received five injections of cocaine (10 mg/kg) or saline, one injection per day. Animals were allowed to habituate to the locomotor chamber for 30 min, after which they were injected with saline or cocaine and then placed back into the chamber for 45 min. Behavioral sensitization was assessed after a drug-free period of 7 days by injection of a low dose of cocaine (1 mg/kg). The low dose of cocaine was chosen to avoid the development of stereotyped behaviors that could interfere with cocaine-induced locomotion in sensitized mice.

#### Cocaine-Induced Conditioned Place Preference

A randomized unbiased CPP procedure was used. CPP chambers were rectangular in shape and consisted of two compartments (19.5 x 19.5 x 30 cm) separated by a guillotine door. One compartment had a smooth floor with white walls and vertical black stripes (GRID-), while the other had a grid floor and black walls (GRID+). Video tracking software (Viewpoint) was used to determine time spent in each of the compartments.

On the pre-conditioning day, mice were free to explore the entire apparatus for 15 min. The time spent in each compartment was recorded and considered a measure of spontaneous preference. Mice that spent more than 70% of the time in one of the two compartments were excluded from the study. Regardless of phenotype and sex, the group average time in one of the compartments during the pre-conditioning day did not deviate significantly from the duration expected on the basis of chance (e.g. 50%).

Conditioning sessions were performed using a randomized unbiased experimental design over 4 or 8 days, with morning and afternoon sessions separated by at least 4 h. During these sessions, mice were randomly injected with either saline or cocaine (10 or 20 mg/kg) and immediately confined to one of the assigned pairing compartments for 30 min. The order of treatments (saline or cocaine), the time of cocaine injection (morning or afternoon), and the compartment (GRID- or GRID+) were counterbalanced. Control mice received saline in both compartments.

On the test day, animals were given free access to the entire apparatus. The time spent in each compartment was measured during 15 min and the preference scores were calculated by subtracting the time spent during the pre-conditioning day from the time spent during the test day in the cocaine-paired compartment. For the control groups, the paired compartment was chosen arbitrarily given that there was no natural preference for either compartment.

### Western Blotting

Twenty-four hours after the last conditioning session, mice were decapitated and their brains were serially sliced in 240 µm-thick coronal sections. The dorsal striatum (dStr) and nucleus accumbens (Acb) were sampled on three sections and removed using 1.50 and 1.20 mm diameter punches, respectively.

Frozen tissues were homogenized in 10 μL of homogenization buffer containing a modified Radio-Immuno Precipitation Assay (RIPA) buffer [100 mM Tris-HCl pH 7.8, 300 mM NaCl, 2% Nonidet P-40, 1% sodium deoxycholate and 0.2% sodium dodecyl sulfate (SDS), 1% EDTA 0.5M] and protease inhibitors (Halt Protease Inhibitor Cocktail, ThermoScientific) using an ultrasonic processor. After centrifugation (14 000×g for 20 min at 4°C), the supernatant was boiled at 95°C for 6 min for protein denaturation (0.06 M Tris-HCl pH 6.8, 2% SDS, 10% glycerol, 0.025% bromophenol blue, 5% β-mercaptoethanol).

Protein concentration was determined using a Bradford assay. Forty μg of protein (diluted in 4X sample buffer) was loaded into 4–12% gradient Bis-Tris polyacrylamide gels for electrophoresis fractionation (Bio-Rad Laboratories) at 200 V for 40 min. The proteins were transferred onto a polyvinylidene fluoride (PVDF) membrane (0.45 µm) at 90 V for 40 min. Membranes were cut at the level of 37 kDa according to protein markers (Precision PlusProtein, All Blue Standard, Bio-Rad Laboratories) and blocked in 3% non-fat Dry Milk (Bio-Rad) for 1 h.

The upper membrane (>37 kDa) was incubated with rabbit anti-actin antibody (1:20 000, A5060 Sigma Aldrich) and the lower membrane (<37 kDa) was incubated with rabbit anti-BDNF antibody (1:500, sc-546 Santa Cruz Biotechnology) at 4°C overnight. The two membranes were incubated for 1 h at room temperature with a horseradish peroxidase-linked anti-rabbit secondary antibody (1:20 000; Bio-Rad Laboratories) and revealed using a chemiluminescence detection kit (ECL Plus). The bands were scanned (Chemioluminescence imager, CIQLE, University Lyon 1) and analyzed using ImageJ software (National Institutes of Health). Actin was used as an internal loading control and each sample was standardized to its corresponding value.

### Fos Protein Immunohistochemistry

Two hours after the CPP test, mice were deeply anesthetized with sodium pentobarbital (50 mg/kg, i.p.) and intracardially perfused with 0.9% saline followed by 4% paraformaldehyde (PFA) in 0.1 M phosphate buffer (PB). The brains were removed, immersed overnight in 4% PFA in 0.1 M PB, and placed in 30% sucrose/PB for cryoprotection. Free-floating coronal brain sections (30 µm) were processed using a standard avidin-biotin-peroxidase method as previously described (Colussi-Mas et al., 2007). Primary antibodies were rabbit anti-Fos raised against a peptide located at the N-terminus of the human Fos protein (1:4 000; sc-52, Santa Cruz Biotechnology). Immunostained sections were analyzed using a light microscope (Axioskop) equipped with a motorized X-Y sensitive stage and a CCD digital camera (Lumenera Infinity 2) connected to a computerized image analysis system (Mercator, ExploraNova). The planes of the analyzed sections were standardized according to Paxinos and Franklin’s (2001) mouse brain atlas. Fos immunoreactive neurons were quantified as previously described (Colussi-Mas et al., 2007). Representative photomicrographs of Fos-positive nuclei in the nucleus accumbens were acquired using an AxioScan Z.1 slide scanner (Zeiss).

### Statistical Analysis

Normally distributed and homogeneous data from behavioral experiments were evaluated by a parametric two-way analysis of variance (ANOVA) followed by a posthoc Newman-Keuls for multiple comparisons. Immunohistochemistry and Western blotting data did not meet the assumptions of normality; therefore, they were analyzed by a non-parametric Kruskal-Wallis test followed by a Mann-Whitney U-test for pairwise comparisons. A probability level of 0.05 or smaller was used to indicate statistical significance. Statview 5.0 software (Abacus Concepts) was used for these statistical analyses. A 95% confidence interval was calculated to compare ED_50_ values.

## Results

### Sensitivity to Acute Cocaine Psychomotor Effects

**Figure 1.**
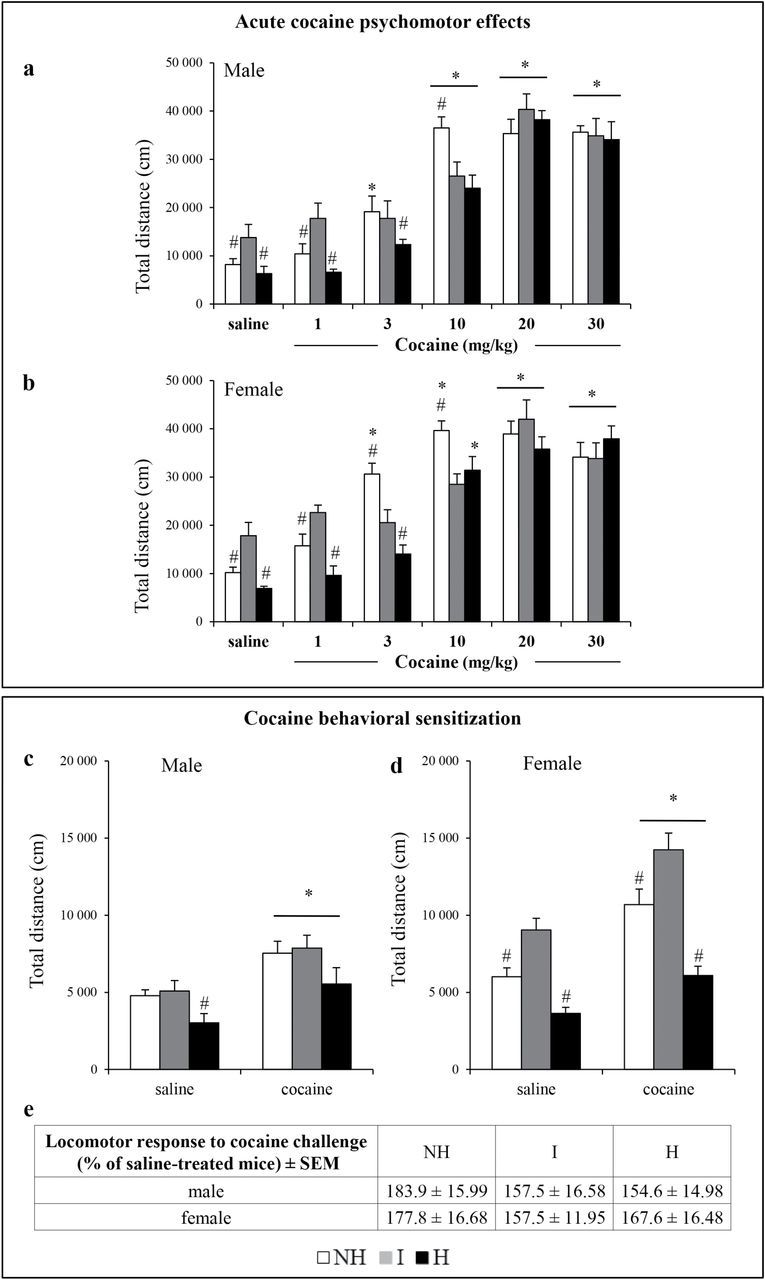
Psychomotor stimulant effects of acute and repeated cocaine injections in male and female mice of the different mouse lines. Total locomotion over 45 min after an acute injection of saline or cocaine at different doses (1, 3, 10, 20, or 30 mg/kg i.p.) are plotted in (A) male and (B) female mice of the three mouse lines: non-helpless (NH), intermediate (I), and helpless (H; n = 8–12). Expression of cocaine behavioral sensitization is depicted as the mean distance traveled over 15 min after a challenge injection of cocaine (1 mg/kg i.p.) in mice that have been saline- or cocaine-pretreated; measurement was performed after a drug-free period of 7 days in (C) male and (D) female mice of the three mouse lines (n = 8–9). (E) Percentage changes in locomotor activity after the cocaine injection challenge (1 mg/kg i.p.) in cocaine-treated mice compared to respective saline control mice (n = 8–9). Data are expressed as mean + standard error of the mean (SEM). *Significant differences from respective saline control (*p*<0.05). #Significant differences from I mice (*p*<0.05).

To determine whether a depressive-like state affects sensitivity to cocaine psychomotor effects, dose-response curves for cocaine-induced locomotor activity were performed (Figure 1A and B) and ED_50_ values were estimated in all mouse lines of both sexes.

As expected, cocaine injections increased locomotion when compared to saline-treated mice. Two-way ANOVAs revealed a significant interaction of treatment and line both in male [F_(10,171)_ = 2.42, *p*<0.05; Figure 1A] and female [F_(10,187)_ = 4.90, *p*<0.001; Figure 1B] mice. Interestingly, analyses of the dose–response relationship highlighted different minimal effective cocaine doses (from 3 mg/kg in male and female NH mice to 20 mg/kg in female I mice) but revealed an identical maximal effective cocaine dose (20 mg/kg).

Consistent with these observations, the estimated ED_50_ values for the psychomotor effects of cocaine indicated that male NH mice were more sensitive to cocaine (ED_50_: 3.32±0.51 mg/kg; *p*<0.05 vs I) than male I mice (ED_50_: 9.97±1.51 mg/kg). In male H mice the ED_50_ value (6.85±2.98 mg/kg) was lower than in male I mice, although the difference did not reach statistical significance.

In female mice, the ED_50_ value for the psychomotor effects of cocaine significantly increased from 1.78±0.82 mg/kg in NH (*p*<0.05 vs I) to 4.3±2.36 mg/kg in H (*p*<0.05 vs I) and to 10.12±1.3 mg/kg in I mice (*p*<0.05 vs H and NH).

When injected with saline, the locomotor response was lower in H and NH mice compared to I mice for both sexes [male: F_(2,32)_ = 4,379, female: F_(2,38)_ = 13,798; #*p*<0.05 compared to H; Figure 1A and B].

### Mice Similarly Sensitized to Cocaine Psychomotor Effects

To investigate whether repeated cocaine injections induce similar neuroadaptations in the brain circuits that mediate its psychostimulant effects, we examined cocaine behavioral sensitization in the different mouse lines. Repeated cocaine administration during the induction phase of sensitization did not change the locomotor response to cocaine, whatever the sex and the mouse line (data not shown). However, as shown in Figure 1C and D, all mouse lines developed a behavioral sensitization when challenged with cocaine (1 mg/kg) after a drug-free period of 7 days. Indeed, cocaine-pretreated mice exhibited significantly higher locomotor responses to the challenge cocaine injection than did saline-pretreated mice of both sexes [male: treatment effect F_(1,43)_ = 43.761, *p*<0.001; female: treatment effect F_(1,46)_ = 19.157, *p*<0.001; *p<0.05 cocaine vs saline-treated mice of the corresponding line]. The magnitude of the increase in locomotor response was not statistically different between the three male and female mouse lines (Figure 1E), suggesting that neuroplastic changes that developed in neural systems mediating cocaine psychomotor effects were similar in all mouse lines.

It is worth noting that the locomotor activity induced by the challenge cocaine injection in saline-conditioned mice (Figure 1C and D) was lower than that of mice receiving a single cocaine injection (Figure 1A and B). This is likely due to habituation to the locomotor activity chamber, handling, and injection procedure for 5 consecutive days before the challenge cocaine injection in saline-conditioned mice.

### Mouse Phenotype Affects Cocaine-Associated Contextual Memory in a Sex-Specific Manner

**Figure 2.**
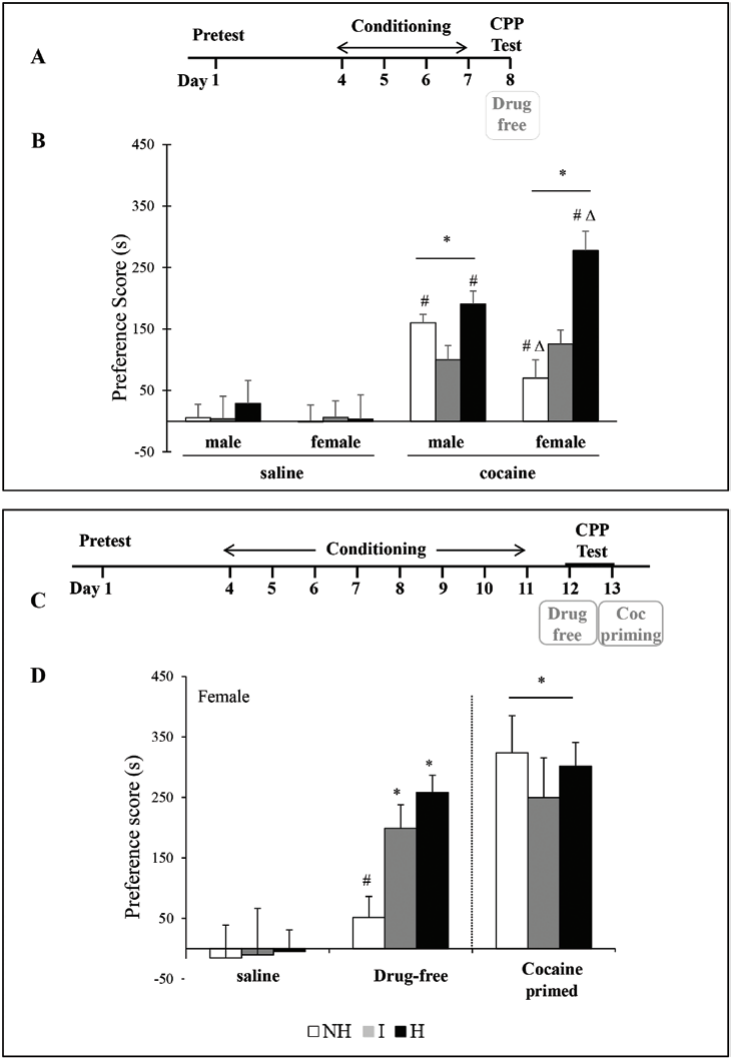
Cocaine-induced conditioned place preference (CPP) in the different mouse lines. (A) Four-cocaine and four-saline conditioning sessions were performed and mice were tested for CPP 1 day after the last conditioning session (day 8), while drug free. (B) Expression of cocaine-induced CPP, as measured by preference scores, was investigated during a 15-min CPP session (in s + standard error of the mean [SEM]) both in male and female mice of the 3 mouse lines (n = 7–14). (C) Eight cocaine and eight saline conditioning sessions were performed and mice were tested for CPP 1 day after the last conditioning session while in a drug-free state (drug-free day 12), and 2 days later under cocaine-primed condition (cocaine-priming day 13; a 10 mg/kg cocaine injection was given immediately before the CPP test). (D) Preference scores observed during a 15-min CPP session (in s + SEM) in females of the three mouse lines under drug-free and cocaine-primed conditions (n = 4–9). Data are expressed as mean + SEM. *Significant differences from respective saline control mice (*p*<0.05). #Significant differences from I mice (*p*<0.05). Δ Significant differences between male and female mice within the same mouse line (*p*<0.05). H, helpless; I, intermediate; NH, non-helpless.

To assess whether a depressive-like state affects the expression of cocaine-associated contextual memory, CPP tests were conducted and preferences scores were evaluated 1 day after four cocaine pairings (10 mg/kg; Figure 2A, data in Figure 2B).

During the pre-conditioning period, no statistical difference was observed in the time spent in the two compartments between the three mouse lines, regardless of phenotype, sex, and treatment (data not shown).

In male cocaine-treated mice, a two-way ANOVA revealed a significant effect of treatment [F_(1,58)_ = 51.31, *p*<0.001] and line [F_(2,58)_ = 3.048, *p* = 0.05]. Posthoc analyses revealed that all mouse lines expressed a significant preference for the compartment previously paired with cocaine, but NH and H mice had significantly higher CPP scores than I mice (*p*<0.05; Figure 2B).

In female cocaine-treated mice, a two-way ANOVA identified a significant interaction of treatment and line [F_(2,56)_ = 6.08, *p* = 0.004]. H mice displayed robust preference scores for the cocaine-paired compartment, resulting in a significantly greater CPP magnitude compared to I and NH mice (*p*<0.05; Figure 2B). The CPP magnitude in I mice was also higher than in NH mice (*p*<0.05).

Comparison of CPP scores in male and female mice of the three mouse lines indicated a significant interaction of sex and line 1 day after the last conditioning session [F_(2,66)_ = 7.93 *p*<0.001]. Female H mice exhibited higher CPP scores than male H mice (*p*<0.05). Conversely, female NH mice displayed lower CPP scores than male NH mice (*p*<0.05). These results indicate that phenotype had a different impact on cocaine-induced CPP in male and female mice; female H and NH mice behaved significantly different in the CPP paradigm when compared to corresponding male mice. Similar CPP scores were observed when mice of both sexes were conditioned with 20 mg/kg of cocaine (data not shown).

Control groups of mice that received only saline injections during conditioning showed no preference on the test day.

### Cocaine-Associated Contextual Memory is Acquired Faster in Female Depressive-Like H Mice

We focused on female mice to better understand the striking differences between mouse lines observed in cocaine-induced CPP scores (Figure 2C, data Figure 2D).

After extension of the conditioning period (8 days instead of 4), the magnitude of CPP scores increased in I mice (*p*<0.05) and reached those of H mice. These findings suggest a faster acquisition of the cocaine-induced CPP in female H mice. The CPP scores remained unchanged in NH mice (data Figure 2D, left panel).

In order to evaluate whether the absence of CPP in NH mice was linked to impaired cocaine-context association or reduced drug rewarding effects, we administered a priming cocaine injection (10 mg/kg) immediately before the CPP test, performed 2 days after 8 days of cocaine conditioning. NH mice had enhanced CPP scores during the cocaine-primed session as compared with their previous scores under drug-free conditions (*p*<0.001) and displayed a preference for the cocaine-paired context similar to that of I and H mice (Figure 2D, right panel). These data indicate that cocaine-context association rather than cocaine rewarding effects was deficient in NH mice.

### Cocaine Conditioning Increases Striatal and Accumbal BDNF Levels Only in Depressive-Like H Mice

To further understand the enhanced response to cocaine-associated context observed in female depressive-like H mice, we investigated potential variations in BDNF levels. We compared BDNF levels in the dStr and Acb in saline-treated mice 24 h after 4 days of cocaine conditioning. The 24 h time point was selected because the three mouse lines expressed different cocaine-induced CPP scores (Figure 2A, data Figure 2B).

**Figure 3.**
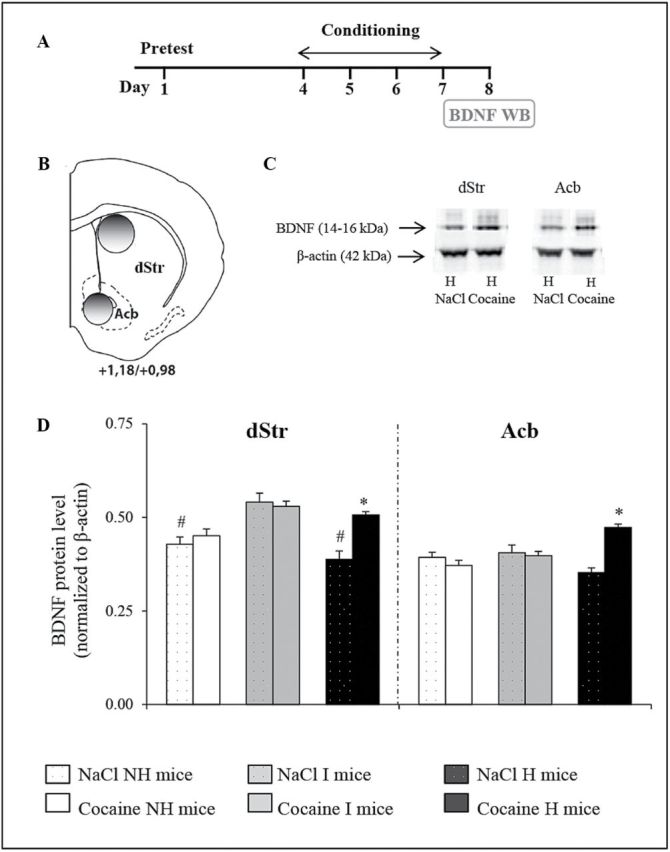
Effect of cocaine conditioning on striatal and accumbal brain-derived neurotrophic factor (BDNF) levels. (A) Female mice were subjected to four-cocaine and four-saline conditioning sessions but did not perform the conditioned place preference test. These mice were killed 24 h after the last conditioning session (day 8) for BDNF Western blotting (WB). (B) Schematic representation of the location of punches for BDNF protein level quantification (Bregma +1.18/+0.98, adapted from the Paxinos and Franklin [2001] mouse brain atlas). (C) Representative immunoblots showing BDNF and β-actin levels in saline- and cocaine-conditioned depressive-like H mice. (D) BDNF protein levels (normalized to β-actin for sample loading control) in cocaine- and saline-conditioned mice of the three mouse lines (n = 8–12). Data are expressed as mean + standard error of the mean. *Significant differences from respective saline control (*p*<0.001). #Significant differences from I mice (*p*<0.001). Acb, nucleus accumbens; dStr, dorsal striatum; H, helpless; I, intermediate; NH, non-helpless.

In saline-treated mice, BDNF levels were lower in the dStr of NH and H mice compared to I mice (*p*<0.001), but there were no differences in the Acb. Interestingly, cocaine conditioning significantly increased BDNF levels to the same extent (around 35%) in the dStr and Acb of depressive-like H mice (*p*<0.001), but not of NH and I mice (Figure 3D).

### Depressive-Like State in Female H Mice Alters the Neuronal Activation Profile Elicited by Retrieval of Cocaine-Associated Contextual Memory

**Figure 4.**
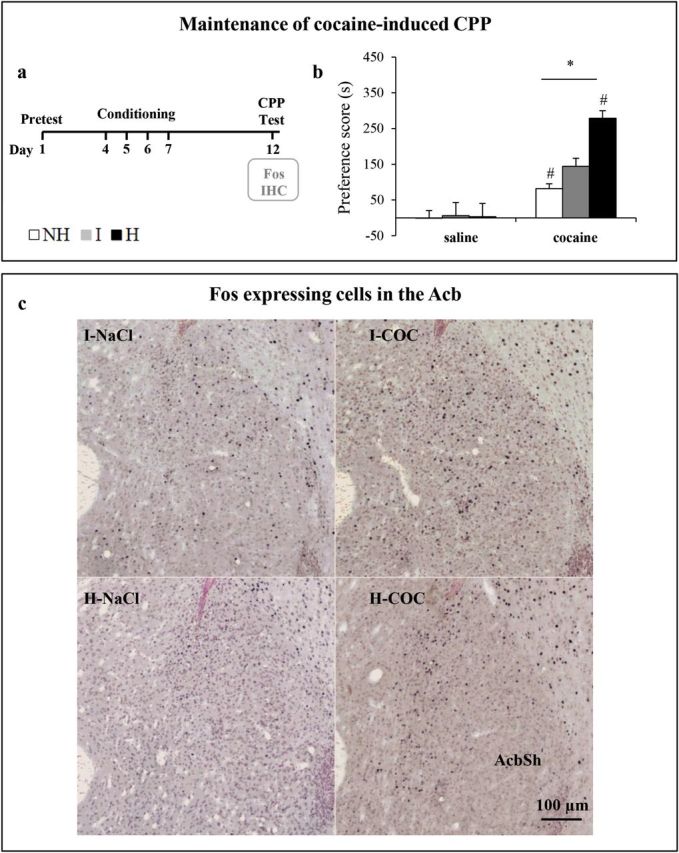
Fos-expressing cells in the nucleus accumbens of female mice during retrieval of cocaine-associated contextual memory. (A) Four-cocaine and four-saline conditioning sessions were performed and female mice were tested for conditioned place preference (CPP) 5 days later (day 12) while in a drug-free state. Mice were perfused 2 h after the CPP test and the brain used for Fos immunohistochemistry. (B) Expression of cocaine-induced CPP, as measured by preference score, was investigated during a 15-min CPP session (in s + standard error of the mean) in female mice of the three mouse lines (n = 7–14). (C) Representative photomicrographs of Fos-positive nuclei on neutral red-counterstained coronal sections in the shell of the nucleus accumbens (AcbSh) of saline- and cocaine-conditioned H and I mice perfused 2 h after the CPP test. *Significant differences from respective saline control (p<0.05). #Significant differences from I mice (p<0.05). Acb, nucleus accumbens; NaCl, (saline); COC, (cocaine); H, helpless; I, intermediate; NH, non-helpless.

**Figure 5.**
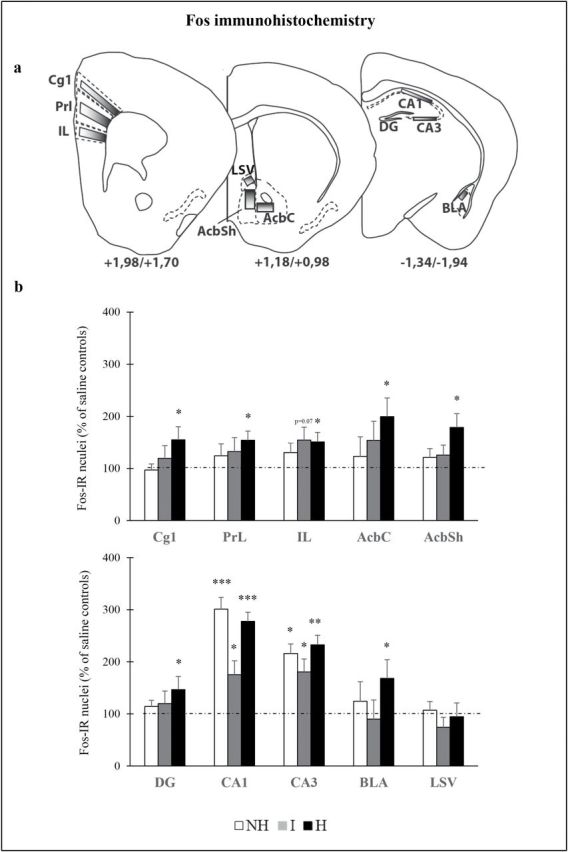
Neuronal activation profile elicited by retrieval of cocaine-associated contextual memory in female mice. (A) The diagrams adapted from the Paxinos and Franklin (2001) mouse brain atlas indicate the placement of grids for counting. Bregma +1.98/+1.70: Cg1, cingulate cortex 1; PrL, prelimbic cortex; IL, infralimbic cortex. Bregma +1.18/+0.98: AcbC, core of the nucleus accumbens; AcbSh, shell of the nucleus accumbens; LSV, ventral part of the lateral septal nucleus. Bregma −1.34/-1.94; DG, dentate gyrus; CA1, CA1 region of the dorsal hippocampus; CA3, CA3 region of the dorsal hippocampus; BLA, basolateral amygdala. (B) Effect of 4 days of cocaine conditioning on Fos expression 2 h after a 15-min conditioned place preference test performed 5 days after the last cocaine conditioning session (Figure 4A; n = 6–9). Percentage of Fos-expressing cells/mm^2^ in cocaine-conditioned female of the three mouse lines compared to corresponding saline-conditioned mice. The dotted lines on the graphs represent the mean for each saline mouse line. Data are expressed as mean + standard error of the mean. Significant differences from respective saline control: *p<0.05, **p<0.01 and ***p<0.001. H, helpless; I, intermediate; NH, non-helpless.

To ensure the maintenance of cocaine-induced CPP, we performed the CPP test 5 days after the last conditioning session (Figure 4A, data Figure 4B). In addition, we explored how recent memory of cocaine-associated context was retrieved in the female mouse lines by comparing the pattern of forebrain-activated regions during the CPP test. We used Fos expression as a marker of neuronal activation (Figures 4C and 5A, data Figure 5B).

As shown in Figure 4B, all female cocaine-conditioned mice displayed a preference for the cocaine-associated compartment [two-way ANOVA, significant interaction of treatment and line F_(2,47)_ = 9.97, *p*<0.05]. However, female H mice achieved higher and more robust CPP scores when compared to I and NH mice (*p*<0.05; Figure 4B), as previously observed 24 h after the last conditioning session (Figure 2B). The CPP scores were not statistically different from those observed 24 h after four conditioning sessions.

Analysis of Fos expression showed that in basal conditions (i.e. in saline-conditioned mice; Supplementary Table 1), the densities of Fos-expressing cells were lower in H mice compared to I mice, specifically in the prelimbic cortex (PrL), shell of the nucleus accumbens (AcbSh), and basolateral amygdala (BLA; *p*<0.05).

In depressive-like H mice that expressed high CPP scores, exposure to the cocaine-associated context induced a significant increase in Fos expression in subsets of neurons distributed in subdivisions of the medial prefrontal cortex (mPFC) (cingulate cortex [Cg1], PrL, infralimbic cortex [IL]; around 50%, *p*<0.05) and several subcortical regions (core of the nucleus accumbens [AcbC], AcbSh, BLA, and dentate gyrus [DG], up to 100%, *p*<0.05; Figure 4C and 5B). In contrast, exposure to the cocaine-associated context in I and NH mice resulted in a paucity of reactivity or significantly lower reactivity in the aforementioned regions that was particularly striking in the Cg1, AcbSh, and BLA (Figure 4C and 5B). Comparable induction of Fos was observed in the CA1 and CA3 regions of the dorsal hippocampus in the three mouse lines (up to 200%). Surprisingly, only a few Fos-expressing cells were observed in the dStr, the ventral tegmental area, and the orbitofrontal cortex, which are implicated in cocaine-related memories.

Locomotion did not contribute to CPP-induced Fos expression, as no significant differences in the locomotor activity of cocaine- and corresponding saline-conditioned mice were observed during the CPP test (data not shown). These findings suggest that enhanced Fos expression occurred in neuronal populations not directly involved in locomotor activity, but rather in selective neuronal populations encoding the retrieval of learned associations between environmental context and cocaine effects.

## Discussion

An important feature of the enduring vulnerability to drugs is that intense associative memories develop between drug effects and environmental contexts. Thus, encountering environments previously associated with drug use often evokes memories of the drug, induces craving, and precipitates relapses in humans. In the present study, using an original genetic mouse model of depression—the Helpless H/Rouen (H) mice—we have provided data indicating that depressive-like states in females can promote cocaine-associated contextual learning by facilitating the link between the drug effects and its associated environmental context. In particular, we demonstrated that cocaine-associated contextual learning in the CPP paradigm was stronger and developed faster in depressive-like female H mice than in control I mice, a facilitatory effect that was associated with a rapid increase in striatal and accumbal BDNF levels. In addition, we showed that retrieval of cocaine-associated contextual memory in mice re-exposed to the CPP apparatus was linked to a greater Fos activation in restricted subsets of neurons distributed in cortical (Cg1) and limbic (Acb and BLA) regions in female H mice than in the other mouse lines.

One of our main findings highlights the robust and opposite impact of the mouse phenotype, helpless and non-helpless, on female mouse behavior in the CPP paradigm. Indeed, female depressive-like H mice displayed a strong and sustained preference for the cocaine-associated context compared to female I mice, while female NH mice displayed a low preference for the cocaine-associated context. Line differences in cocaine-induced CPP scores were weaker in male mice. Male NH and H mice displayed similar preferences for the cocaine-associated context that were only slightly higher than that of male I mice. These data suggest that affective states (depressive-like vs. non-depressive-like phenotype) in females have more influence on vulnerability to the cocaine-associated context than in male mice. We did not examine the estrous cycle in female mice. However, given the short length of the mouse estrous cycle (4–5 days) and duration of the CPP experimental procedure (8–12 days), as well as findings from a large number of studies we have performed independently, it is unlikely that different levels of circulating ovarian steroid hormones were a major factor favoring contrasting CPP scores in female H mice (Anker and Carroll, 2011).

Mouse line differences observed in the CPP scores are not due to differential sensitivity to the psychomotor or rewarding effects of cocaine for the following reasons. First, the ED_50_ values for acute cocaine psychomotor effects were not directly related to the magnitude of CPP scores. For example, female NH mice exhibiting a high sensitivity to cocaine psychomotor effects displayed low CPP scores. Next, behavioral sensitization, induced by repeated cocaine exposure, such as during cocaine conditioning, developed similarly in the three mouse lines. Finally, the CPP scores, at least in female mice, were similar in the three mouse lines when tested after cocaine priming injections, suggesting that it is the learned cocaine-context association rather than the effect of the drug that differs between the different mouse lines. In line with this notion, we showed that the impaired ability to form cocaine-context associations was particularly clear in NH mice, since both interoceptive and subjective cocaine effects and cocaine-associated context are required to elicit approach behavior towards this drug (Le Merrer et al., 2011).

That phenotype is a contributor to cocaine-associated con- textual learning is supported by our findings showing that depressive-like female H mice displayed facilitated acquisition of cocaine-associated contextual memories compared to female I and NH mice. These differences are most likely due to an increased initial rate of learning in female H mice. Studies focusing on such an aspect of learning in animal models of depression are lacking. Previous studies using models of depression based on environmental challenges showed that depressive-like states enhanced psychostimulant-induced CPP and operant responses for addictive drugs (Covington and Miczek, 2001, 2005; Nader et al., 2012; Bardo et al., 2013). However, the behavioral mechanisms underlying such interactions remain unclear. Only Whitaker et al. (2013) showed that social isolation in mice, a stress-based procedure that induced a depression-related phenotype, enhanced the initial rate of learning of amphetamine-associated context. These results and our data suggest that increased acquisition of drug-associated memories may represent one of the mechanisms contributing to higher vulnerability to psychostimulants in depressive-like states.

In a first attempt to assess the molecular mechanisms underlying the faster acquisition rate in female depressive-like H mice, we examined BDNF protein expression in the Acb. By virtue of its interconnections with limbic forebrain circuits, encompassing the BLA, hippocampus, and mPFC (Brog et al., 1993; Britt et al., 2012), and its afferents from the ventral tegmental complex (Van Bockstaele and Pickel, 1995; Ikemoto, 2007), the Acb can be considered as a node within the neural circuits underpinning motivation (Wise, 2004) and reward- and aversive-based learning (Bromberg-Martin et al., 2010; Hikida et al., 2010). Dysfunctions within the Acb are implicated in both depression and drug use disorders. In addition, the molecular alterations shared by these two disorders were recently shown in rodents to involve the gene encoding the neurotrophic factor BDNF (see Russo and Nestler, 2013, for review). Quite unexpectedly, we found no notable change in accumbal BDNF levels in naive female depressive-like H mice, indicating that the increase in accumbal BDNF signaling observed in stress-induced models of depression (Weiss et al., 2001; Krishnan et al., 2007; Bessa et al., 2013) should not automatically be extended to genetic animal models of depression. This observation strengthens the notion that the molecular signature of depression caused by environmental stressors may differ considerably from that caused by genetic predisposition (Malki et al., 2014).

Pertaining to cocaine use vulnerability, investigation of BDNF expression uncovered a clear-cut increase in accumbal and striatal BDNF levels in female H mice 24 h after cocaine conditioning. In the Acb, this result contrasts with results from earlier studies performed on common strains of adult rodents in which a sustained time-dependent increase in BDNF protein expression was observed only several days after passive or self-administered cocaine injections (Grimm et al., 2003; Huang et al., 2011; Li et al., 2013). The lack of accumbal BDNF changes in NH and I mice 24 h after cocaine exposure is in agreement with these data. The substantial increase in BDNF observed in female H mice, however, indicates a facilitated induction of BDNF by cocaine in depressive-like mice. A rapid up-regulation of BDNF expression in female H mice may drive the faster acquisition of the cocaine-induced CPP. This notion is supported by previous studies showing that stimulation of BDNF-TrkB (Tropomyosin Receptor Kinase B) signaling in the Acb during cocaine conditioning strongly potentiates cocaine-induced CPP (Bahi et al., 2008; Graham et al., 2009). The crucial role of accumbal BDNF as a positive modulator of psychostimulant-related behaviors was also observed in drug self-administration and relapse procedures (Graham et al., 2007; Bahi et al., 2008; McGinty et al., 2010; Walsh et al., 2014). Concerning the dStr, we found a substantial decrease in BDNF levels in naive female H mice as compared to I control mice, and a strong increase only in cocaine-conditioned female H mice compared to respective saline control mice. Interestingly, a previous study evidenced a substantial enhancement in striatal BDNF levels after extended, but not restricted, access to cocaine self-administration and a positive role for this neurotrophic factor in cocaine intake (Im et al., 2010). Although our study did not directly address the contribution of altered accumbal and striatal BDNF levels to the facilitation of cocaine-induced CPP in the depressive-like female H, it suggests that cocaine generates molecular events, such as enhanced accumbal and striatal BDNF expression, which differentially occur in depressive-like states and might engender increased vulnerability to psychostimulant drugs. This issue warrants further investigation.

Regarding the retrieval of cocaine-associated contextual memory in mice re-exposed to the CPP apparatus in the absence of cocaine, we identified several neuronal populations distributed in the AcbSh, Cg1, and BLA (also to some extent the DG and AcbC) that were more Fos reactive in depressive-like female H mice. Other areas, such as the CA1 and CA3 regions of the dorsal hippocampus, did not show differential activation. The anatomically-selective impact of the depressive-like phenotype on brain reactivity to a cocaine-associated context emphasizes a higher reactivity of brain regions critically involved in the formation and retrieval of associations between the drug effects and the environmental context (BLA; Everitt et al., 1991) and their translation into motivated behaviors (Acb and mPFC; Stefanik and Kalivas, 2013; Lalumiere, 2014), rather than those involved in encoding spatial and contextual information independently of their emotional valence (dorsal CA1 and CA3 regions of the dorsal hippocampus; Fanselow and Dong, 2010; Zelikowsky et al., 2014).

Our data are relevant with regard to previous human and animal studies that have consistently implicated structural and functional alterations in the above mentioned limbic-cortical-striatal regions after cocaine exposure (Koob and Volkow, 2010; Stuber et al., 2010 for reviews) or in depressive disorders (Krishnan and Nestler, 2008; Russo and Nestler, 2013 for reviews). However, these studies did not directly investigate the possible link between these alterations and the greater vulnerability to psychostimulant drugs observed in depressed patients or depressive-like rodents.

Collectively, our anatomical data highlight the neuronal plasticity initiated by cocaine-conditioning in a limbic-cortical-striatal network that differs between depressive-like H mice and their controls. Although our study did not test the functional significance of enhanced Fos activation in this network, it provides a foundation for further exploring how this network acts to shape the high sensitivity to cocaine-associated context in depressive-like states.

In conclusion, our results fuel the emerging interest in the neurobiological mechanisms underlying comorbidities of depression and drug use disorders. Our original data identify relevant alterations in learning mechanisms, accumbal BDNF expression, and cortico-striato-limbic circuit reactivity that could mediate enhanced cocaine vulnerability in depressive states. Furthermore, our finding emphasizes the importance of characterizing sex differences in depressive- and drug-related pathways in order to understand the heightened predisposition of females towards these disorders.

## Supplementary Material

For supplementary material accompanying this paper, visit http://www.ijnp.oxfordjournals.org/

## Statement of Interest

The authors report no biomedical financial interests or conflicts of interest.

## Supplementary Material

Supplementary Table 1
