# Supplementary material for: Enhanced Cocaine-Associated Contextual Learning in Female H/Rouen Mice Selectively Bred for Depressive-Like Behaviors: Molecular and Neuronal Correlates
Source: Int J Neuropsychopharmacol. 2015 Apr 30;18(8):pyv022. doi: 10.1093/ijnp/pyv022 (PMC4571631; doi:10.1093/ijnp/pyv022)
Supplement: Supplementary Table 1 [file Rappeneau_Supplementary_Table_1.docx]

| **Brain areas** | **Fos-expressing cells/mm^2^ in saline-conditioned mice** | | |
| --- | --- | --- | --- |
|  | NH/Rouen | I/Rouen | H/Rouen |
| Cg1 | 133 ± 16.70^##^ | 83 ± 11.70 | 67 ± 7.90 |
| PrL | 185 ± 26.12^#^ | 176 ± 27.39 | 107 ± 16.37^#^ |
| IL | 245 ± 26.35^##^ | 197 ± 29.90 | 149 ± 20.76 |
| Acb core | 50 ± 13.49^###^ | 30 ± 8.44 | 11 ± 1.76 |
| Acb shell | 230 ± 33.01^###^ | 172 ± 27.30 | 93 ± 15.05^#^ |
| BLA | 226 ± 57.63^#^ | 219 ± 47.99 | 115 ± 24.21^#^ |
| LSV | 605 ± 49.23 | 481 ± 81.02 | 640 ± 57.97 |
| DG | 252 ± 23.58^#^ | 179 ± 23.45 | 158 ± 18.43 |
| CA1 | 273 ± 88.16 | 210 ± 58.52 | 168 ± 22.78 |
| CA3 | 171 ± 48.86 | 209 ± 42.63 | 172 ± 19.08 |
